# Supplementary material for: Transient Hepatitis B Surface Antigenemia Following Immunization with the Adjuvanted Hepatitis B Vaccine Fendrix®
Source: Vaccines (Basel). 2025 Nov 30;13(12):1216. doi: 10.3390/vaccines13121216 (PMC12737611; doi:10.3390/vaccines13121216)
Supplement: Supplementary file 1 [file vaccines-13-01216-s001.zip › vaccines-3985238-supplementary.pdf]

ID	sex	age	HBsAg_pre	Anti-HBs_pre	Anti-HBc_pre	DateSerolPre
1	M	79	0,34	<10	0,1	1/3/23
2	M	54	0,29	<10	0,1	9/3/23
3	M	57	0,29	<10	0,2	9/3/23
4	F	76	0,31	<10	0,1	17/5/23
5	M	80	0,28	<10	0,1	17/5/23
6	M	68	0,31	<10	0,1	17/5/23
7	M	80	0,38	<10	0,1	17/5/23

date_vac	schedule	HBsAg_post1	Anti-HBs_post1	Anti-HBc-post	HBV-DNA_po	DateSerolpos
2/5/23	Booster	2,37	non-reactive	0,1	not detected	3/5/23
10/5/23	Primary_Cycle	3,11	non-reactive	0,1	not detected	11/5/23
8/5/23	Primary_Cycle	4,62	non-reactive	0,2	not detected	11/5/23
23/5/23	Booster	4,69	non-reactive	0,1	not detected	30/5/23
30/6/23	Booster	3,06	non-reactive	0,1	not detected	30/5/23
24/5/23	Booster	2,36	non-reactive	0,1	not detected	26/5/23
24/5/23	Booster	1,37	non-reactive	0,1	not detected	30/5/23

HBsAg_post 2	Anti-HBs_pos	Anti-HBc_pos	HBV-DNA pos	DateSerolPost	HBsAg_post3	Anti-HBs_pos
1,37	non-reactive	0,1	not detected	5/5/23	0,28	non-reactive
0,37	non-reactive	0,1	N/A	27/5/23	N/A	N/A
2,4	non-reactive	0,2	not detected	27/5/23	0,4	non-reactive
0,3	non-reactive	0,1	N/A	26/6/23	N/A	N/A
0,27	non-reactive	0,1	N/A	26/6/23	N/A	N/A
0,32	>1000	0,1	N/A	10/7/23	N/A	N/A
0,34	non-reactive	0,1	N/A	12/6/23	N/A	N/A

Anti-HBc_pos	HBV-DNA_po:	DateSer_post3
0,2	not detected	12/5/23
N/A	N/A	N/A
0,1	not detected	20/6/23
N/A	N/A	N/A
N/A	N/A	N/A
N/A	N/A	N/A
N/A	N/A	N/A
